# Cold Stress, Freezing Adaptation, Varietal Susceptibility of *Olea europaea* L.: A Review

**DOI:** 10.3390/plants11101367

**Published:** 2022-05-20

**Authors:** Raffaella Petruccelli, Giorgio Bartolini, Tommaso Ganino, Samanta Zelasco, Luca Lombardo, Enzo Perri, Mauro Durante, Rodolfo Bernardi

**Affiliations:** 1 Institute of BioEconomy, National Research Council (CNR/IBE), 50019 Sesto Fiorentino, Italy; raffaella.petruccelli@ibe.cnr.it (R.P.); giorgio.bartolini@ibe.cnr.it (G.B.); 2 Department of Food and Drug, University of Parma, 43124 Parma, Italy; 3 Council for Agricultural Research and Economics-Research Centre for Olive, Fruit and Citrus Crops, 87036 Rende, Italy; samanta.zelasco@crea.gov.it (S.Z.); luca.lombardo@crea.gov.it (L.L.); enzo.perri@crea.gov.it (E.P.); 4 Department of Agricultural, Food and Agro-Environmental Sciences, University of Pisa, 56121 Pisa, Italy; mauro_durante@yahoo.it (M.D.); rodolfo.bernardi@unipi.it (R.B.)

**Keywords:** olive tree, cold injury, physiological processes, molecular processes, cultivar tolerance

## Abstract

Olive (*Olea europaea* L.) is an evergreen xerophytic tree characterizing vegetative landscape and historical-cultural identity of the Mediterranean Basin. More than 2600 cultivars constitute the rich genetic patrimony of the species cultivated in approximately 60 countries. As a subtropical species, the olive tree is quite sensitive to low temperatures, and air temperature is the most critical environmental factor limiting olive tree growth and production. In this present review, we explored the detrimental effects caused of low temperatures on olive cultivars, and analyzed the most frequently experimental procedures used to evaluate cold stress. Then, current findings freezing stress physiology and gene are summarized in olive tree, with an emphasis on adaptive mechanisms for cold tolerance. This review might clear the way for new research on adaptive mechanisms for cold acclimation and for improvement of olive growing management.

## 1. Introduction

Olive (*Olea europaea* L.) is an evergreen xerophytic tree characterizing vegetative landscape and historical-cultural identity of the Mediterranean Basin. Olive domestication probably occurred in the eastern regions of the Mediterranean, through vegetative propagation and diffusion of trees with favorable morphological and agronomic characters [1,2,3]. The “proto-farmers” probably selected olive trees with particular traits such as bigger fruits, higher oil content or adaptation to environmental and anthropic conditions. The desirable genotypes were preserved, multiplied by vegetative propagation and transferred in new growing areas. The spreading of the olive culture throughout the Mediterranean Basin was promoted by human migrations and commercial exchanges, then a local process of selection and diversification occurred generating a large number of varieties that still characterize the olive rural landscapes today. Accordingly, more than 2600 cultivars constitute the rich genetic patrimony of the species [4] even if only 250 varieties are classified as “commercial cultivars” by the International Olive Oil Council [5]. Olive is estimated to be cultivated in approximately 60 countries, with an olive growing area of over 11 million hectares, for the predominant part (95–97%) located in Mediterranean area [4,6,7]. However, an expansion in olive cultivation has been occurring in non-traditional countries such as Australia, Argentina, Chile, China, Ukraine (Crimea), Japan and the United States. Geographically, olive trees are traditionally cultivated between the 30th and 45th parallel, both in the northern and southern hemisphere. Recently, olive cultivation has been expanding in regions at lower latitudes, like in the foothills of the Andes mountains (27–33° S latitude) and in some provinces of China like Yunnan, Guizhou or Guangxi (22–27° N latitude in a monsoon climate) [8,9,10,11]. A single cultivation was found beyond the 50th parallel N in South-West England, in Devon [12]. Regarding the altitude, olive trees can be grown from sea level up to generally 700–800 m above sea level [13]. Nevertheless, in Mediterranean (Italy, Spain, Morocco, and Lebanon), and non-Mediterranean (China, Peru, and Argentina) countries, olive is also cultivated at higher altitudes, from 900 to 1200 m a.s.l., [9,14,15]. and in the Atlas Mountains in North Africa can survive or grow up to 1600–1700 m [16,17].

**Figure 1:**
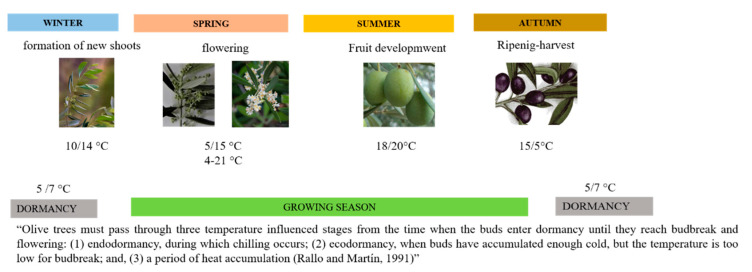
Temperature ranges for the species in relation to the thermal requirements of the various phenological phases according to the BBCH scale.

For the environmental conditions, olive is considered one of the most suitable and best adapted species to the Mediterranean-type climate, characterized by mild and humid winters, with minimum temperatures of about −4 °C, and hot and dry summers, with maximum temperatures of about 50 °C; and most of the rainfalls concentrated in the cold season. [16,18,19,20]. Under the climate conditions of Mediterranean region, the vegetative-reproductive cycle is biennial in which production and induction of flower buds coexist at the same time, on the growing shoot, for fruiting the following year [13,21,22,23,24,25] (Figure 1).

These growth phases differ according to cultivars and are controlled by different environmental factors, and the temperatures of the coldest (temperature of January) and warmest months (mean temperature of July) are the meteorological parameters that most influences different phases of the phenological cycle of the olive tree, such as foliation, flowering or fruit ripening [26,27,28,29,30].

Notwithstanding, excessively low temperatures and recurrent winter frosts can systematically appear in particular areas of Italy, Spain, France and Portugal (but also South America, Australia and China) where the olive tree is grown [31,32]. In this regard, historical sources reporting severe cold damage in olive groves date back to the 12th up to the 17th century. More accurate information is available for the intense anomalous winter temperatures that occurred between the eighteenth and the twentieth centuries. In particular, the frosts occurred on February in 1929, 1956 and 1985 caused a marked olive production decline following the death of many olive trees (Table S1). It has also been estimated that, in Mediterranean area, the risk of freezing for olives occurs at irregular intervals between 10 and 40 years [13,32,33]. In more recent years, cold damage and production failure occurred in 2016 and 2018 [34,35]. In a context of climate change, simulation models have been revealing a steady increase in the global average temperature, but this uptick is frequently accompanied by irregular and extreme cold events, even if of short duration (from 24 h to a few days) [36,37]. The increase in mean temperatures might allow to extend olive cultivation at more northern latitudes, where low temperatures are currently its main limiting factor [38]. On the other hand, the frequent returns of cold that we have been witnessing in recent years are affecting the productivity of olive groves even in some traditional regions. For these reasons frost-tolerance in olive has gained relevance in new breeding programs and/or varietal selection. In spite of this, low temperature (LT) has not been well documented as other abiotic stresses (e.g., drought) in olive. In fact, from the literature search conducted in the ISI Web of Science (WoS) for the 2000–2021 period, using the keywords “stress and olive tree” and “cold stress and olive tree”, about 360 articles were found, but little research had a reference to the low temperatures.

This review provides a complete overview of the current knowledge on impact of low temperatures on olive phenology, morphology and physiology, cold responses and adaptation mechanisms, behavior of olive cultivars in relation to frost stress and agronomic management in order to provide exhaustive information useful for new breeding program and mitigation/contrast strategies in an era of climate change.

## 2. Low Temperature Stress in Olive Tree

**Figure 2:**
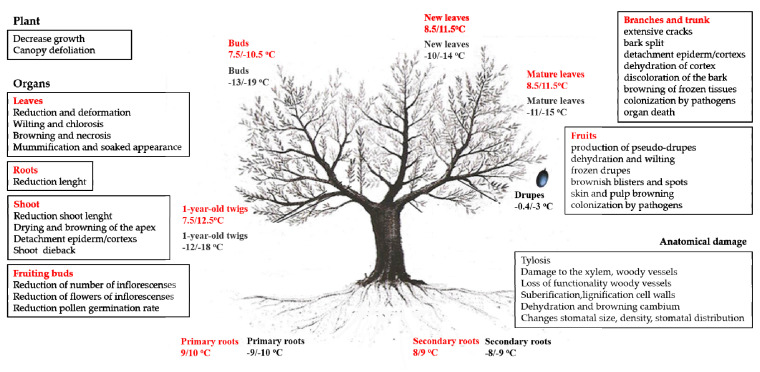
Critical (in red) and freezing (in black) temperatures in different organs of the olive tree [12,16]. In the box, major damage suffered by olive tree as a result of cold stress.

The optimal temperature for olive growth and development is around 20–30 °C. At a sub-optimal temperature (10/20 °C) it undergoes a rapid decrease in production; at chilling-temperature or critical temperature (from 7.5 to 12.5 °C) are strongly slow down the metabolic processes such as respiration and photosynthesis; at freezing temperatures (temperatures average below −10 °C) irreparable damage to organs and tissues and the death of the entire plant can occur. In ecological and agronomic contexts, it is very useful to know the critical temperature and freezing temperature that play a decisive role in tree phenology and adaptation (Figure 2) [16,31,33,39,40]. In spite of a high ecological plasticity of the species, there are few observations on the vegetative-productive behavior of a cultivar transferred from warm to cooler zones. The temperature thresholds for frost damage in olive trees depend on the phenological phases of the plant and on the season in which low temperatures occur (early frost, winter frost and late frost). Generally, at minimum temperatures of 0 °C/−3 °C (slight damage) injury is observed to the younger organs of the tree (leaves, one-year shoots) with reduction of growth and production; at minimum temperatures of −6/−7 °C (moderate damage) almost all the organs of the plant are damaged; at temperatures below −12 °C/−18 °C (severe damage) the whole plant can be compromised [31,33,41,42,43,44,45]. These studies suggest that olive can withstand temperatures below −8 °C for a relatively long period of time (for more than one week), while can tolerate freezing temperatures only for a short time (hours) and it is capable of dealing with temporary and transient alterations induced by LT. Furthermore, the effects of intensity of cold temperatures are conditioned by plant age (young plants are more susceptible than older plants), the hardening or dehardening phase and by differential survival of their specific organs and tissues [15,16,29,31,33,46].

Cold stress in plants is classified in “Chilling stress” and “Freezing stress”. Chilling stress refers to low, but not freezing, temperatures (usually between 0 °C and 15 °C), instead freezing stress occurs when temperature drops below 0 °C [47]. Chilling stress is less harmful to plants when compared to freezing stress. Both these stresses have direct as well as indirect effects on plant growth, development, metabolism, and consequently in reducing of crop production.

### 2.1. Chilling Stress

In chilling-sensitive plants (i.e., subtropical and tropical plants) low temperatures (<12 °C) can be detrimental to all phases of plant development, causing phenotypic symptoms (i.e., decrease in plant growth, reduction of roots length, reduction of leaf expansion, loss of leaves, chlorosis and necrosis) and damage on reproductive organs (reduced pollen production and viability, reduced pollen tube growth and increased flower abortion) [48]. In addition, chilling temperatures cause metabolic and structural dysfunctions such as alteration in respiration and photosynthetic activity, protein synthesis and in the properties of the membranes with consequent changes in the activity of the enzymes, decrease in membrane permeability, ion-solute leakage and cellular dehydration [49,50,51]. Chilling temperatures also, affect all components of water system causing loss of water and an evident dehydration of the tissues [48,52]. Despite the subtropical origin of *Olea europaea* L., chilling temperatures do not irreversibly compromise the development of plant and visible injuries occur under very LT. It has been reported that the response to cooling temperatures (0/2.2 °C) varies in relation to the sensitivity and/or tolerance of the different olive cultivars: visible symptoms are evident in 1–2 weeks in susceptible plants and in 6–7 weeks in tolerant cultivars [13].

Temperatures below 4 °C, or around 0 °C, markedly compromise plant growth, productivity, and cause a delay in flowering. Furthermore, the reproductive organs, flower buds, flowers and fruit are seriously damaged [29,53,54,55]. The exposure of plants to low temperatures (below 10 °C) induces changes in the function of the photosynthetic apparatus, causing photoinhibition phenomena [56] changes in respiration and enzymatic activity [33,40] and alteration in the plasma membrane potential (V_m_) [39]. Low temperatures also cause dehydration symptoms in shoots and leaves due to an imbalance between water absorbed by roots and transpiration [57,58]. Relations between chilling temperatures and water parameters has been evaluated both in field condition and potted plants [58,59,60,61]. Lopez Bernal et al. [61] evaluated the influence of low winter temperatures on the water parameters (water potential, ψ, stomatal conductance, Gs and hydraulic resistance R_root_) of mature olive trees (cv Arbequina). The authors observed a marked increase of root hydraulic resistance (R_root_) leading a reduction of leaf water potential (ψ_leaf_ and ψ_stem_) and stomatal conductance, confirming the cold-sensitiveness of the root system. Pavel and Fereres [59] in 1-year-old plants (cv Picual) and Perez-Lopez et al., [60] in 6 cultivars (Cornicabra, Picual, Arbequina, Ascolana Tenera, Frantoio and Changlot Real) identified two soil chilling temperatures at which the plants exhibited water stress. At the soil temperatures below 10 °C and below 6.4 °C the plants exhibited a slight and/or severe decline of water parameters, while stomatal conductance decreased as the soil temperature dropped to 6.4 °C. Soil-chilling temperatures cause dehydration stress due to an imbalance between the absorption of water by the root and transpiration. The authors suggested that the reduction in root absorption may be related to a general reduction in root activity or the increase in water viscosity at lower temperatures. The response of the cultivars to soil chilling may be used as an indicator of their frost resistance [60]. The acquisition of chilling tolerance in olive is poorly understood as only a few studies have been published [33,40,62]. Rejŝková et al. [62] investigated, under in vitro conditions, the changes in the soluble carbohydrate fraction in shoot segments (cv Picual) exposed chilling temperatures (4 °C). The authors observed an increase of total endogenous carbohydrate content; in particular, changes were observed in the proportions of mannitol and raffinose family oligosaccharides (RFO), while the sucrose proportion did not change. Recently, Mougiou et al. [40] analyzed the response of Koroneiki and Mastoidis cvs subjected to mild cold stress (LT 0 ± 2 °C). The cold stress treatments conducted for a few days did not affect nor physiological (photosynthetic rate, stomatal conductance and intercellular CO_2_), biochemical parameters (antioxidant enzymes activity) neither plants phenology. Koroneiki and Mastoidis cvs were classified as tolerant to mild cold stress [40]. This behavior was confirmed by analysis of genes involved in hydroxytyrosol biosynthesis (olive polyphenol oxidase, OePPO; tyrosine decarboxylase, OeTDC; alcohol dehydrogenase, OeALDH; and copper-amine oxidase OeCuAO). The authors showed that all genes upregulated their expression levels as a response to chilling temperature in both cultivars.

### 2.2. Freezing Stress

Severe frost temperatures (below the freezing point of water) cause substantial decline in the metabolic efficiency and physiological processes. Often, these changes can be irreversible, potentially leading to plant death. The damage caused by freezing is the formation of ice crystals in organs and tissues of the plants. The formation of ice crystals causes a decrease in the apoplastic water potential, due to the chemical potential difference between aqueous solution and ice, and subsequently attracts water from the inside, resulting in cellular dehydration. Freeze-induced dehydration is responsible for various forms of physiological and metabolic changes [63,64]. In relation to the location of ice formation, two types of freezing are recognized: intracellular freezing (water freezes inside the cell) and extracellular freezing (water freezes outside the cells, in the intercellular spaces). The formation of ice in the extracellular spaces is due to the lower freezing point of intracellular aqueous solution and to the presence of heterogeneous cryogenic agents (bacterial proteins, dust etc.). Intracellular ice formation, predominantly caused by rapid freezing stress, leads a “mechanical disruption of the protoplasmic structure” which inevitably leads to the death of cells [47,63]. Ice formation inside or above an organ depends on the rate at which the cells are cooled, on nucleation temperature of ice formation (INT) (crystallization temperature) and presence of ice nucleation active (INA, bacteria and non-bacterial sources). INT is the temperature at which the first ice crystals form in tissues. It has been determined that plants with cryogenic agents (INA, bacteria and non-bacterial sources), ice formation occurs in the range of −2 and −5 °C; while in plants with scarce presence or absence of cryogenic agents, INT is between −5 and −10 °C. For example, the average temperature that catalyzes the formation of ice is around −2 ° C in peach tree, −1.9 ° C and −2.1 °C in apple and pear respectively and −6.4 °C in Citrus [65,66].

Surico and Lavermicocca [67] identified the presence of ice nucleating agents on shoots of three different olive cultivars (Nostrana, Coratina and Leccino), and determined the temperature that catalyzes the formation of ice (INT) ranging from −2.7 °C to −5.5 °C. The authors hypothesized that the distribution of ice nuclei inside or above olive shoots, may be irregular and that the ice nucleating agents can promote frost damage to plants tissues and subsequently, they can cause indirect damage such as favoring the onset of pathogens infections (e.g., *Pseudomonas savastanoi pv. Savastanoi*). Recently, Arias et al. [68,69,70] determined INT in leaves, shoots and roots of young olive plants (1–2-year-old plants) of the cvs Arbequina, Changlot Real, Frantoio, Hojblanca and Manzanilla. The authors observed differences in ice nucleation temperatures (INT) during winter among cultivars and organs analyzed; in particular, the temperatures recorded for the roots were −2.1 °C in Arbequina and −6.3 °C in Frantoio, for stems were −5.77 °C in Manzanilla and −10.84 °C in Frantoio and −6.07 °C in Manzanilla and for leaves −11.89 °C in Frantoio. Fiorino and Mancuso [31], hypothesized that ice formation occurred *in primis* in the stem, due to the larger vessels, containing more water, and subsequently may rapidly propagated to leaves. Larcher [71] determined threshold temperatures inducing 50% freezing lethality (LT_50_; the temperature at which half of the plants die because of freezing injury) in different organs of *Olea europaea* L. The LT_50_ values resulted to be −12 °C for leaves, −16 °C for twig cambium and xylem, −8 °C for buds and −6 °C for root cambium. At moment, different LT_50_ indices of leaves, stems, buds and roots are reported in literature determined by several analysis methods (i.e., electrolyte leakage, differential thermal analysis, visual score, electrical resistance) and by degree of freezing tolerance (FT) of several cultivars. All data were obtained through laboratory tests under controlled conditions. Fiorino and Mancuso [31], and Arias et al. [68] reported that order of sensitivity in the different organs of olive was: secondary roots > primary roots > apical leaves > basal leaves > shoots > vegetative buds. In olive trees, two different types of buds have been identified, the principal (primary) and the accessory (secondary) bud [24,72]. The primary bud is more susceptible to freezing injury than accessory buds. Therefore, a freezing condition may only affect the primary buds and not the secondary ones. The latter could represent a “center for renewed growth” allowing the plant to recover and growth.

### 2.3. Morphological and Anatomical Consequences of Frost Damage

**Figure 3:**
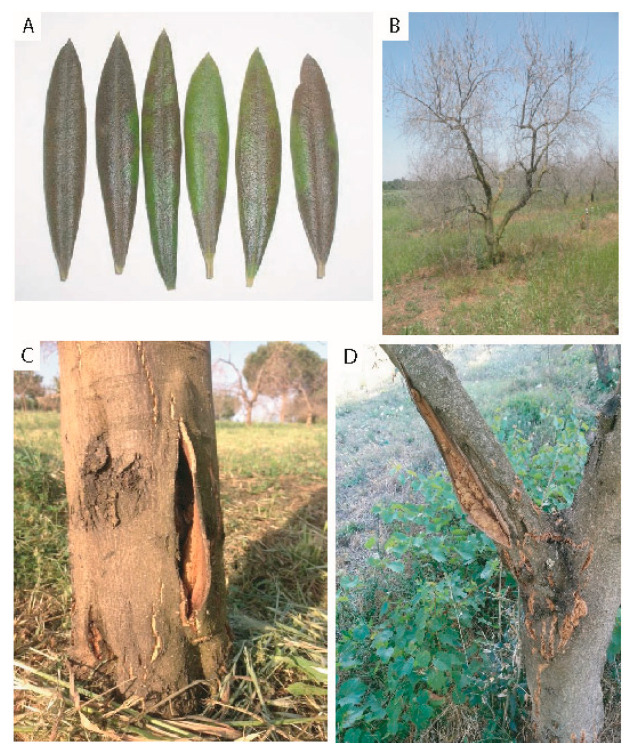
Symptoms of freezing stress: (**A**) leaves chlorotic and brown; (**B**) total defoliation; (**C**) extensive cracks of trunk; (**D**) extensive cracks of branches. The photos were kindly provided by V. Sergeeva and G. Pannelli.

Visible symptoms of plants exposed to freezing has been reported by several authors [19,73,74,75,76]. Leaves being one of more susceptible organ to cold stress, already at −2/−3 °C show damages leading to defoliation, at −10 °C leaves become chlorotic and brown, mummified, deformed and soaked, subsequently (after also 20–30 days) they dry up and fall off (Figure 3A,B). Severe freezes (below −10 °C) may lead to necrosis of the leaf apex that after can be colonized by secondary saprophytic bacteria and fungi. Frequency or intensity of defoliation can reduce root growth, respiration, nutrient uptake and formation and development of flowers, resulting in severe yield losses [33,44,75,77]. The further decrease in temperatures (<−15 °C) determines the extension of damage to the shoots up to the bark. The vegetative apexes of the shoots (usually the first 4–5 nodes) darken and subsequently die; the 1–2-year-old branches show cracks and detachment of the epidermis from the bark; the main branches and the trunk show extensive cracks, necrosis of large areas of the cortex and its subsequent detachment (Figure 3C,D). Cracking causes an immediate loss of water with consequent dehydration of the cortex and death of the affected organs. Discoloration of the bark and wood may also be observed due to the rupture and loss of function of the vessels. The browning of frozen tissues is probably due to the oxidation of phenolic compounds poured into the cell walls when the cell membrane is destroyed by ice crystals [33,73,77,78,79,80]. Damage to root tissues was observed when soil temperature reached −2 °C [68]. Low temperatures (<−3/−4 °C) during spring can cause serious damage to flowers and inflorescences, reduce pollen germination and the number of flowers per inflorescences, and stimulate the formation of pseudo-drupes [13,33,72,77,78]. A drop in temperature during fall, (from −0.4 °C to −3 °C) before harvesting, can induce dehydration and wilting of the drupes, the presence of surfaces with brownish blisters and spots, completely frozen drupes, internal browning around the pit, skin browning and slight discoloration of flesh [19,75,81,82], and reduction in the accumulation of oil [11,13,79]. Furthermore, the small lesions caused by low temperature can be colonized by pathogens and modify the oil quality, oil stability and sensory changes conferring defect to the oil (“frozen oil”) [83,84]. Cold stress affects, also, anatomical structures such as stoma, mesophyll tissue, and epidermal cells. Cross-sections of freeze-damaged leaves highlighted the death of many cells containing the green chloroplasts in the area near the vascular bundle and the destruction of palisade parenchyma and epidermis [79].

Cold temperatures cause limited water absorption while transpiration is maintained in a steady state. Under these conditions, stomatal size, density, and pattern of stomatal distribution in leaves are affected. Tylosis, death of cambial cells, suberification and lignification of the cell walls and damage to the woody vessel were observed few weeks after the freezing event [77,85,86]. The cambium may dehydrate or turn brown, due to the breakdown of cells. In young plants of Picual and Ascolana cvs were observed separations between phloem and sclerenchyma, between old and new xylem, and breaks in the phloem tissue. Death of cambial cells, suberification and lignification of the cell walls were observed in the stem [46,79,86], while, deposition of tannins and/or rubbery substances was observed in parenchymal cells (cortex or pith zones) and xylem vessel [46,78,79,87,88].

### 2.4. Physiological and Biochemical Consequences of Cold Stress

Photosynthetic metabolism is among the most sensitive processes to temperature fluctuations, and the exposure to low temperatures limits the photosynthesis causing the disruption of all the major components of the process, such as inhibition of electron transport photophosphorylation, reduction in the maximum quantum efficiency of Photosystem II (PSII), photochemistry, carbon fixation. PSII is one of the most susceptible components of the photosynthetic machinery and the chlorophyll fluorescence parameter Fv/Fm is a non-invasive measurement of PSII activity and is a commonly used technique to monitor the responses of plants to environmental change [88,89,90]. Several authors, under LT conditions, observed also in olive decreases in the levels of net photosynthesis rate, changes in transpiration and stomatal conductance, damage of PSII and alterations in photosynthetic pigments highlighting notable differences among olive cultivars. Changes in Fv/Fm ratio has been observed under freezing temperatures. Tolerant genotypes were reported to maintain a significantly higher Fv/Fm index, while a decline was observed in non-tolerant CVs, indicating malfunction or damage to PSII reaction centers [91,92,93,94,95,96]. Reduction of photosynthetic activity accompanied by soluble sugar accumulation as well as hydrolysis of starch in chloroplasts, probably caused by disturbances in carbohydrate metabolism or by an inhibition of sugar utilization, were found in the leaves of several cultivars [45,68,74,97,98]. LT may also cause loss of cellular turgor, disturbances in plant water relations and decrements in leaf water potentials [58,68,69,94]. Alterations in concentrations of total soluble proteins (TSP), and in photosynthetic pigments are reported in Amphisis, Gorgan, and Manzanilla cvs [94], in Frantoio and Sevillana cvs [99], and in Leccino and Oblica cvs [100]. LT induces oxidative stress, due to overproduction of reactive oxygen species (ROS): superoxide radical (O_2−_), hydrogen peroxide (H_2_O_2_) or a hydroxyl radical (HO-) [52]. Excess production and accumulation of ROS cause protein oxidation, lipid peroxidation of membranes and accumulation of Malondialdehyde (MDA), breaking up of polysaccharides, enzyme inhibition, destruction of pigments and DNA damage [101,102]. Oxidative damage caused by ROS under artificial low/freezing temperatures has been reported in Fishomi and Roughani [103], Leccino and Oblica [100], and Askolyana (Ascolana), Razzo and Correggiolo cvs [104]. One the final products of membrane lipid peroxidation, Malondialdehyde (MDA) accumulation at higher concentrations in sensitive genotypes was observed; [94,104]; MDA is considered a biomarker for oxidative damage [105]. In addition, under cold stress occur accumulation of secondary metabolites (e.g., anthocyanin and phenolic compounds) [106,107,108], and changes in hormones biosynthesis and distribution [109]. Under LTs some olive cultivars showed changes in total phenolic compounds, oleuropein, and proline content [96,103,104,107].

The bio-membrane systems (plasma membrane -PM-, cell membrane, nuclear membrane and organelle membrane) are considered the primary site of damage during exposure to cold stress [47,110]. It has been established that the formation of ice crystals causes mechanical damage (i.e., membrane rupture, distortion of cellular shape due to extracellular ice formation, deformation of cell walls and deformation of plasma membrane due, presumably, to adhesions of ice with cell-wall polymers) and cellular dehydration, altering the permeability of PM. Visually, these alterations appear as a cell flaccidity, water-soaked appearance and / or discoloration of injured tissues [111,112]. The physiological result is an enhanced intracellular solute concentration due to dramatic electrolyte loss (predominantly K^+^ and a small, but significant loss of Ca^2+^) [113]. Under low temperatures D’Angeli et al. [114] observed, a transient increases in cytoplasmic free calcium [Ca^2+^]_cyt_ in leaf protoplasts (Leccino, Frantoio, and Moraiolo cvs), caused both by Ca^2+^ efflux from the organelles and Ca^2+^ influx through the plasma membrane. Other authors observed the same phenomenon in leaf and drupe protoplasts [115,116]. Freezing alters significantly the stabilization and fluidity of biomembranes. Such changes may result from changes of membrane lipid composition (under LT the percentage of unsaturated fatty acids increases), distribution ratio of lipids, lipid protein ratios, and changes in membrane structure from a liquid-crystalline phase to a solid gel phase [63,111,113]. It has been observed that cold-sensitive plants usually have a higher proportion of saturated fatty acids in their plasma membrane, while cold-resistant plants have a higher proportion of unsaturated lipids, because of their lower melting temperature [52]. The reduction of permeability and increased of membrane viscosity severely affect the membrane transport, reducing the absorption of water and the transport of sugars [117]. The several changes in cellular processes induced by low temperatures can be ascribable to a non-adaptive response, simple expression of damage, and/or adaptive responses that act as signals or sensors that activate protective mechanisms in plants (e.g., cold-induced tissue damage repair, rebalancing of cellular homeostasis, modulation of growth) [117]. In olive cultivars, the study of cellular changes of metabolite pools or structural components may serve to identify the variations in response to freezing stress and to discriminate between frost-tolerant and frost-sensitive olive cultivars.

## 3. Responses, Adaptions and Recovery at Low Temperature

Plants use two different adaptation strategies to withstand or survive cold temperatures: freezing avoidance and/or freezing tolerance [47]. Mechanisms of freeze avoidance are associated with adaptations and structural aspects of a plant that impact ice formation. Supercooling process is a prevalent mechanism of freeze avoidance in which water remains in the liquid state at sub-zero temperature, preventing intracellular freezing and limiting cell dehydration. Mechanisms of freezing tolerance (cold hardening), instead, affect physiological, biochemical, and epigenetic changes that allow to the plant response to cold stress [64,118]. It is evident that tolerance and avoidance are not mutually exclusive categories; the same plant can draw on different strategies from those based on “avoidance” to those of “tolerance” to counteract or to withstand freezing temperatures [119,120]. In autumn, when the temperatures become sub-optimal for growth (between 5/10 °C) and the photoperiod is reduced, olive trees block their vegetative growth reaching the stage of dormancy. A series of physiological and metabolic changes occur leading to the cold acclimation process and prepare themselves physiologically to low temperature tolerance. It is widely recognized that cold acclimation is induced or strengthened by LT, short photoperiod or their interaction, and is a species/cultivar-specific process (22]. Recently, López-Bernal et al. [121] reported that in olive tree the exposure to low temperature, rather than the photoperiod, is the primary environmental signal that triggers the cessation of vegetative growth and the cold acclimation. The plant easily loses cold acclimation when winter temperatures are mild. Indeed, unseasonal warm episodes (1–2 weeks at 16 °C) can cause “premature de-acclimation” resulting in a loss of tolerance to frost and risk of damage to the de-acclimated tissues when the temperature drops again [122,123,124]. To counteract the adverse effect of cold stress olive trees exploit both supercooling mechanism and freezing tolerance. The effectiveness of these mechanisms depends on many factors, as well as the speed, the rate and the degree of temperature decline, the climate conditions antecedent cold temperature injuries, the stage of acclimation, the cultivar and the genetic capability of tissues to adapt to freezing stress. Supercooling strategy in olive has been observed in most of organs, (leaves shoots, buds and roots) by several authors [31,124,125,126]. even if root system (in particular not yet lignified roots), has little aptitude to cold acclimation [31]. The employment of Differential Thermal Analysis (DTA) is a common method to measure freezing damage. A single-peak exotherm (Low Temperature Exotherm—LTE) was in generally found in almost all organs and cultivars evaluated However some contrasting results were found i.e., in leaves) [78,126].

Bartolozzi and Fontanazza [97] evaluated the capacity to supercool of 9 olive cultivars and three selections by DTA. They reported that olive bark tissues did not exhibit supercooling. Bark tissues displayed only the High Temperature Exotherm (HTE; the heat released when super cooled water freezes extracellularly) but did not show any LTE peak. Freezing point of extracellular water in olive bark ranged from −6.3 °C to −8 °C, but no statistical differences among varieties were found. All the values were not indicative of tissue death (extracellular freezing is generally considered nonlethal). DTA technique does not seem suitable in discriminating varietal response to freeze stress. On the contrary, electrolytic leakage (LT_50_ is calculated from this measure) is considered a powerful discrimination method showing a general concordance with visual assessment of damage. Recently, Arias et al. [68,69] investigated the role of apoplastic water, solute content, and cell wall rigidity in freezing avoidance by supercooling mechanism in five olive cultivars. The authors observed that cultivars with lower apoplastic water content, higher solute content and higher cell rigidity showed higher supercooling capacity during the winter. In olive tree, response to freezing stress have been highlighted in several studies mainly focused on physiological and biochemical responses in non-acclimated (NA) and cold-acclimated (CA) plants. All the trials showed that cold acclimation increased frost tolerance in comparison with non-acclimated plants.

### 3.1. Biochemical Responses

Calcium plays an important role during the acquisition of freezing tolerance and [Ca^2+^]_cyt_ constitutes the primary sensing mechanism for low temperatures. Using non-cold-acclimated and cold-acclimated leaf protoplasts. D’angeli et al. [114] evaluated [Ca^2+^]_cyt_ changes in Leccino (cold-tolerant), Frantoio (moderately cold-tolerant) and Moraiolo (cold sensitive). The results indicated that during acclimation the increase of [Ca^2+^]_cyt_ can be significantly attenuated or blocked, suggesting that the attenuation of the calcium response provides a “cold memory”. This behavior was more evident in cultivars with high and/or medium cold tolerance. Furthermore, the close relationship between Ca^2+^ signaling and genotype characteristics for acclimation was highlighted. Furthermore, there are strong evidences showing changes in total soluble sugar (TSS) and total soluble protein (TSP) during frost stress in several cultivars so much so as to suggest that the qualitative-quantitative variation of these compounds could be effectively used for varietal screening in relation to cold tolerance. Carbohydrate metabolism were associated with increase in frost tolerance in various deciduous fruit tree and wood plants [64,65]. Although the specific functions of carbohydrates have yet to be fully understood, they have been shown to stabilize plant cell membranes, preserve water in cells and mitigate the toxicity of reactive oxygen species [64,88]. Preliminary studies of biochemical changes in plant response to low temperatures have been reported by Antognozzi et al. [74] and Bartolozzi et al. [127,128] on Italian olive cultivars. In 1994, Antognozzi et al. [74] detected that water potential, starch and water content decreased with decreasing temperature, whereas soluble sugar increased lead to discriminate among tolerant/susceptible varieties. Other authors confirmed previous results reporting a progressive accumulation of mannitol, sucrose and TSPs during the CA stage in several organs (i.e., leaves, bark) and cultivars, elucidating the regulatory action of sucrose in CA and FT in olive [97,127,128,129,130].

Changes in FT of different olive cultivars were consistent with leaves tissue dehydration (relative water content -RWC-), accumulation of cryoprotectants (namely soluble carbohydrates, proline, phenolic compounds) and scavenging capacity during hardening [96,103,131,132]. Accumulation of unknown polypeptides (66 kDa and 43 kDa in leaves and 70 kDa, 43 kDa and 16 kDa in bark) was also detected during the CA stage [130]. The authors speculated that these polypeptides could belong to the dehydrin-like family, or be cold-resistant membrane proteins. Proteins related to the dehydrin-like family have been found in local Turkish and foreign olive cultivars [129]. The authors detected during cold-acclimation, 43 and 23 kDa polypeptides and reported that the 43 kDa dehydrin was closely related to cold-hardiness (Table S2).

Gulen et al. [98] also observed a higher content of total phospholipid and phospholipid fractions (phosphatidylcholine, phosphatidylethanolamine and phosphatidylinositol) in CA stage and in tolerant cultivars than in NA stage and non-tolerant cvs. The authors proposed that the rise in phosphatidylcholine (PC) levels showed by tolerant plants, was related to the synthesis of the so called cold-regulated (COR) proteins, like dehydrins, and may represent a response to both low temperatures and freeze-induced dehydration. Rahemi et al. [45] monitoring 9 olive varieties, showed higher concentrations of proline, phenolic compounds, reducing sugars, and lower level of starch in frost-tolerant varieties.

At a later time, D’angeli and Altamura [115] investigated. the role of osmotin, a multifunctional stress responsive pathogenesis-related protein 5 (PR-5), in cold response and acclimation in the cv Canino. Using protoplasts of control plants and transgenic plants (containing the tobacco osmotin gene) in CA and NA stages, the authors observed that in non-cold-acclimated plants, the expression of osmotin protein (24 kDa) was present in the transgenic plants only. Control plants, on the other hand, presented the protein only after acclimation and in tissues that showed programmed cell death. Furthermore, in NA control plants changes of [Ca^2+^]_cyt_ were observed. Interestingly, the study results showed the multiple activities of osmotin. In olive trees osmotin is involved in programmed cell death (PCD), which is cold inducible, regulates cytoskeleton alterations and blocks [Ca^2+^]_cyt_ changes. Matteucci et al. (2011) [116] monitoring the changes in [Ca^2+^]_cyt_ in drupes of cultivar differing in cold tolerance (Frantoio semi hardy, Moraiolo non hardy, Canino hardy), observed that cold acclimation was achieved at the completion of oil accumulation and that the response of cold acclimation was genotype-dependent. Indeed, cold acclimation is acquired by the drupes of the cultivar capable of cold acclimation in the leaves, in agreement with the results reported by D’angeli et al. [114].

### 3.2. Antioxidant Responses

Current research has highlighted the close relationship between ROS scavenging and plant stress tolerance; thus, several kinds of antioxidant enzymes are frequently, activated by plants to counter the oxidative stress caused by low temperatures. The most active antioxidant enzymes are catalase (CAT), superoxide dismutases (SOD), peroxidases (PRX), and enzymes in the ascorbate-glutathione cycle. Changes in expressions of many antioxidant enzymes have been observed in genotypes of some cereals and legume growing under cold stress [111,112]. Antioxidant metabolites may protect and stabilize cell membrane and structures and make the plant more tolerant [102].

The first study focused on the role of phenol metabolism in response to a frost event in winter was conducted by Ortega-Garcia and Peragón [107]. After exposure to cold temperatures (−7 °C), the authors selected in field, trees (cv. Picual) with different degrees of damage (from unstressed to heavily cold stressed), and analyzed the activities of phenylalanine ammonia-lyase (PAL) and polyphenol oxidase (PPO) enzymes and the concentration of hydroxytyrosol, tyrosol and oleuropein in olive leaves. The authors documented involvement of PAL, PPO and oleuropein in the response to cold stress. In particular, PPO and oleuropein were related to the antioxidant defense system and PAL was involved in a plant recovery mechanism. The response to cold stress and cold-hardiness of several olive cultivars was also assessed by measuring several other antioxidant enzymatic defense system such as catalase (CAT), ascorbate peroxidase (APX), nicotinamide adenine dinucleotide phosphate (NADPH) oxidase, peroxidase (POD), superoxide dismutase (SOD) in CA and NA stages and in leaves [94,98,104,131,132,133,134,135].

Generally, under cold stress cold-tolerant plants showed increased enzyme activities. In particular, CAT, POD, PPO and NADPH oxidase were directly correlated with the degree of cold-hardiness. Leyva-Pérez et al., [135] observed an overproduction of H_2_O_2_ accompanied by a down-regulation of SOD isoenzymes, a key enzyme of the ascorbate–glutathione cycle (APX) and an antioxidant NADP-dehydrogenase (NADP-ICDH) within 24 h from LT stress treatment. During long-term cold acclimation at 10 days, a slight recovery of Mn-SOD and NADP-ICDH was detected, indicating that a mechanism of cold acclimation takes place. This showed the ability of olive plants to rapidly acclimate to prolonged cold stress.

### 3.3. Molecular Responses

CA acclimation in olive lead to a complex metabolic response which modify the pattern of several compounds. These biochemical and physiological changes are regulated by low temperatures through important changes in gene expression [95,135,136,137,138]. Cold stress response in plants requires crosstalk between multiple signaling pathways including cold, heat, and reactive oxygen species (ROS) signaling networks. CBF, MYB, bHLH, and WRKY families are among the TFs that function as key players in the regulation of cold stress response at the molecular level [139,140].

However, little information is available on the molecular response of olive tree to low temperatures and just a few studies have investigated the molecular mechanisms underlying cold acclimation in olive tree. In a first study, suppression subtractive hybridization (SSH) libraries have been used to characterize Differentially Expressed Genes (DEG) induced by low temperature in mature leaves from two Leccino clones (cold tolerant vs cold sensitive) stressed sequentially as follows: 10 °C for 14 days, 5 °C for 7 days, 0 °C for 1 day, −5 °C for 3 h and −10 °C for 23 h [137]. The sequencing of DEGs- cDNA from the tolerant clone lead to a putative identification of 11 expressed sequence tags (ESTs). The DEGs in response to cold stress codify for proteins involved in several processes such as signal transduction, RNA processing, translation, protein processing, redox homeostasis, photosynthesis, photorespiration, and metabolisms. An expression analysis of the cold gene transcripts was carried out using chloroplast ycf2 protein, bark storage protein, carbonic anhydrase, chlorophyll a,b binding protein, PAL and WRKY probes from mature leaves of plants treated under range of temperatures from 10 to −10 °C. The gene expression profile to different temperatures resulted as biphasic: genes expression level decreased at around 5 °C, then rise up to maximum values at −5 °C. Several examples of biphasic responses are present in different stress treatments. The authors hypothesized a production of phytohormones such as abscissic acid (ABA), salicylic acid (SA), and jasmonic acid (JA), which may control the expression of various genes. These newly synthesized hormones may cause a return or amplification of the initial signals to generate a second round of signaling. Interestingly, in the resistant Leccino clone (18) WRKY was expressed at room temperature but with low intensity, and subsequently increased at low temperatures (0, −5, −10 °C). Opposite behavior was observed in susceptible Leccino clone 4. PAL gene showed a response, during the time-course treatment, similar to those of the WRKY transcript, suggesting an involvement of both the genes in a cold stress resistance response. WRKY belongs to a large transcription factor family, expressed in many different biological processes involving signaling, transcription, chromatin remodeling, and others [141,142]. As known, PAL is involved in the first step of the phenylpropanoid pathway, playing an important role in disease resistance and several developmental and metabolic processes in response to several stress factors.

Moreover, another gene of high relevance is represented by ycf2 a chloroplast functional gene that encodes a protein product, an ATPase essential for cell survival [142,143]. Recently, Kikuchi et al. [144] have elucidated the function of this gene, whose product is involved in the cross-talking processes between nucleus and chloroplast. Chloroplasts import thousands of nucleus-encoded preproteins synthesized in the cytosol and translocated through the TOC/TIC translocon complexes. Preprotein translocation across the inner membrane requires ATP. The import motor is constituted by a 2-MD heteromeric AAA-ATPase complex: the 2-MD complex consists of a protein encoded by the chloroplast gene ycf2 and five related nuclear-encoded FtsH-like proteins. The nucleus/cytoplasmic organelles relationships during cold stress find another interesting example in the expression of the Pentatricopeptide repeat-containing protein complex (PCMP-H11 gene) belonging to the PPR family [137]. Nuclear codified PPR proteins regulate gene expression at the post-transcriptional level in chloroplasts and mitochondria, by editing the RNA sequence, stability, cleavage, splicing and translation.

More recently, RNA-Seq analysis were carried out to monitor the transcriptome changes induced by low temperature in olive [95,135]. Leyva-Pérez et al. [135] stressed 4-month-old potted olive plants of the cv. Picual through long-term exposure to LT. Aerial tissues were harvested at 0 h, 24 h, and 10 days of LT exposure (10 °C day/4 °C night). At the begin of experiment most of plants showed flaccid leaves, resembling wilting symptoms. However, after 4–5 days, plants progressively recovered from these symptoms, until the end of the experiment. For the first 24 h of cold exposure a transient response to cold stress was observed in which 1094 genes were overexpressed and 600 were repressed. Annotated transient transcripts were mostly parent–child related and involved mainly in lipid polymers, oligosaccharide, and polyamine metabolism. During the early long-term LT exposure 204 genes were induced, whereas 594 were repressed. In fact, a general tendency towards a reduction in transcription was observed for most genes. Encoding gene transcripts for several desaturases were found (FAD2.1, FAD2.2, FAD6, FAD7, and two FAD3), but only the oleate desaturase encoding gene (FAD2.2) increased its expression during cold acclimation. A shortly induction at the begin of experiment was detected and high transcript level was maintained after 10 days of LT treatment, suggesting a key role OeFAD2.2 in cold response in olive leaves. Recently, Unver et al. [145] discovered five genes belonging to FAD2 family in oleaster (var. *sylvestris*). From a gene expression analysis conducted in several organs, transcript profile of FAD 2.3 resulted analog to those of FAD 2.2 previously characterized [115,145,146]. The author renamed FAD 2.2 in FAD 2.3. Several studies reported that temperature regulates FAD expression at the transcriptional, post-transcriptional, and post-translational levels [115]. An increase in FAD2 transcript levels have been reported in cotton soybean, Brassica and Ginkgo biloba [147].

The C-repeat binding factor (CBF), transcription factor has been demonstrated to play a pivotal role in several species in cold acclimation. The expression of the CBF genes is induced after an increase of the cytosolic Ca^2+^ level and is responsible for controlling the expression of a large number of genes, so-called Cold Responsive (COR) genes. The CBFs bind to the low-temperature-responsive DNA-regulatory element termed C-repeat/dehydration-response element present in the promoters of many COR genes. In olive transcriptome 4 CBF orthologus were induced by cold in a sustained manner while the low temperature persisted. However, three of them decreased their expression between 1 and 10 days of cold exposure but still remained significantly higher than in control plants. Only OeCBF3 showed a steady increase in its expression after 10 days of cold treatment, suggesting a role for inducing COR gene expression, showing a late response to cold stress. These genes were functionally associated with regulation of gibberellin biosynthesis, cold acclimation, and development regulation. Twenty DEGs were associated with nitrogen metabolism and 34 with photosynthesis. Their expression level reflected in general a down-regulation of photosynthesis and plant growth.

Late long-term gene-expression changes during cold acclimation were also examined. Between days 1 and 10 of LT exposure a general trend of gene repression was observed after a period of 24 h (4.551 genes were down-regulated and only 64 genes were over-expressed). The most representative associated processes were related to organelle fusion, nucleus organization, and DNA integration. Guerra et al. [95], identified the transcriptome changes occurring in leaves of cv. Leccino exposed to a long period of CA by a gradual lowering of temperature in controlled conditions, a treatment simulating as much as possible natural the conditions leading to cold acclimation of olive plants in open field (from 22 °C to −4 °C in 11 about weeks with a freezing step at −4 °C for 5 h). The transcriptome changes induced by low temperatures during a CA treatment in leaves of olive trees cv. Leccino were investigated through a RNA-Seq analysis. Three contrasts were accomplished, namely control vs. partial hardened, partial hardened vs. full hardened, control vs. full hardened, in order to identify short-term modulated genes, long-term modulated genes and genes gradually modulated during the treatment, respectively.

In this work, only one CBF transcript was recovered (one 1121 bp long contig, containing a complete open reading frame; it is most similar to our contig comp32939_c0_seq1 annotated as DREB3-like) and deduced amino acidic sequence revealed the presence of the typical CBF signature. In other species, CBFs are present as multiple copy gene family [148,149,150,151]. The amino acid sequence alignment was conducted both against Arabidopsis CBF sequences and 4 olive CBFs from Picual cv found out by Leyva-Pérez et al. [135] a strong similarity of the olive CBF with dwarf and delayed flowering (DDF) 1 of Arabidopsis and OeCBF2 (few mismatches could be due to intervarietal polymorphisms). OeCBF1, 2, and 4 are 97–98 % similar to each other while OeCBF3 was the most dissimilar one. Since only OeCBF2 contains a complete ORF, while other OeCBFs resulted incomplete and limited to the highly conserved AP2 domain. Based on these last results, the author concludes that so far, only one complete CBF has been identified in olive tree.

In summary, both the RNA-sequencing transcriptome experiments showed an induction of genes encoding enzymes associated to changes in membrane lipid composition, as well as stress-related genes like cold-regulated genes and dehydrins, the downregulation of photosynthesis-related genes, and the activation of the carbohydrate catabolism. The expression pattern of DEGs rather well aligned with previous gene expression studies and biochemical and physiological changes described here and in other species [152,153], suggesting that olive activates known and conserved molecular responses to cold stress. However, Guerra et al., [95] highlighted specific peculiarities of olive tree, such as preferential osmolytes compounds and induced scavenging systems for ROS. Long-term responses during CA seem to be more effective for cultivar screening related to cold-susceptibility [135].

In other species, several genes have been identified to be involved in cold stress response, including genes that encode TFs, phosphatases, and kinases [153]. Genome editing conducted on CBF genes in Arabidopsis through the clustered regularly interspaced short palindromic repeats-associated protein (CRISPR/Cas9) system demonstrated the triple CBF mutants were more at risk of freezing [150]. Thus, indicates CBF genes are critical for cold adaptation, but a number of other FTs with a coordinated action are involved. The gene encoding for OsMYB30 was edited with the CRISPR–Cas9 improving cold resistance in rice. However, further studies (i.e., on CBF/DREB TFs) are needed to increase understanding of applying beneficial strategies to improve plant tolerance to cold stress. Comparative studies with other plant species that already have a wide adaptability to extreme temperature conditions would be necessary to identify cold-induced genes. One example is the work on the Seabuckthorn (Hippophae rhamnoides L., Elaeagnaceae) [154]. This is an important shrubby plant widely distributed throughout Europe and Asia with interesting officinal and nutritional properties; the species is tolerant to environmental abiotic stresses such as drought, salinity and cold. This plant has the capability to survive at temperatures between −40 °C and +40 °C, so it represents a very interesting system if one start from the hypothesis that its genome already has genes predisposed for tolerance to low temperatures. The authors [154] carried out a serial analysis of differential gene expression by using the DeepSAGE technology on control plants (28 °C) and plants under cold (4 °C) and freeze (−10 °C) stress. Three comparative setups, i.e., control vs cold stress, control vs freeze stress and cold stress vs freeze stress, allowed the identification of 428 cold and freeze stress responsive genes: among them genes encoding signal transduction proteins (MAPK kinases) and regulatory proteins (calmodulin-related proteins).

Until few years ago, olive genome sequence was not available and cold-related genes are not currently isolated yet from this species Currently, neither genetic maps nor association studies have been conducted for this complex trait. Recently, more than one olive genome sequence has been obtained [155] and this should accelerate the knowledge and isolation of genes and / or editable allelic variants in favor of assisted breeding and modern biotechnologies in olive.

## 4. Evaluation of Freezing Tolerance in Olive Tree

In general, the studies in *Olea europaea* L. highlighted that acclimation times and capacity are genetically determined but can be modified by environmental factors. Marked genotypic differences were observed by several authors with different methodology and approaches. The main results, methodology in varietal screening were reported in Table S2.

Generally, studies have evaluated cold tolerance of different olive cultivars after a midwinter extreme freeze episode and classify frost tolerant olive genotypes. A subdivision into three classes: (i) tolerant or hardy; (ii), moderately tolerant or semi-hardy; (iii) susceptible or non-hardy, was adopted. Historically, field evaluation of freezing damage by evaluation of plants survival rate after freeze events, has been the first method used to evaluate frost tolerance of olive cultivars. In the field evaluation, due to numerous intrinsic and/or extrinsic factors that influence FT, the results on the degree of tolerance or susceptibility of an olive cultivar may be contrasting and not always generalizable. Moreover, field tests only evaluate the damage reported to the aerial part of the plant, without considering the root part of the tree [156,157]. In olive tree, being an evergreen woody plant able of living for a very long time, an accurate determination of the response to freezing stress and survival is somewhat articulated and complex. In fact, plants can manifest symptoms even several weeks after the freezing event, marked differences occur in the same plant (nearly dead branches and vegetative branches), or among plants placed at a short distance [42,73].

The majority of studies on olive tree used methods based on controlled freezing tests. Freeze test under controlled conditions and several other methods (electrolyte leakage -EL-, differential thermal analysis -DTA-, triphenyl tetrazolium chloride -TC-reduction, electrical impedance analysis, fractal spectrum analysis, chlorophyll fluorescence analysis, stomatal density and biochemical enzymes activity and sugars, phospholipids, proline, phenols) have been used to assess LT_50_ impact on several varieties. EL is an indicator of membrane stability and is the most widely used test to estimate the stress-induced injury in plant tissues and to detect the cold tolerance of plants [111,158,159]. In general, LT_50_ always indicated in all experiments that CA plants had lower LT_50_ values than NA plants and as LT_50_ widely varied in relation to the organ. The cold tolerance screening of olive cultivars was also investigated by analyzing the relationships between stomatal traits and the degree of winter hardiness. The stomata of the olive leaf are only on the abaxial surface and play an important role limiting water loss [29,56]. For example, Roselli et al. [160] and Roselli and Verona [161] reported that low stomatal density and stomatal size correlated with cold hardiness of olive cultivars. The authors indicated that hardy genotypes showed low stomatal density and medium/large size stomata, while the sensitive genotype had high stomatal density and medium size stomata. Stomatal density has been used to classify some Greek olive varieties, and recently, in agreement with the previous reports, the stomatal density allowed to differentiate cold-tolerant from cold-sensitive Iranian cultivars [45,162] (Table S2).

In agreement with Azzarello et al. [156], fractal analysis could be very useful in assessing cold hardiness of plants on the basis of visible injury, without sophisticated or expensive instruments and in a reliable and cost-effective way, using only a scanning device, a personal computer and dedicated freeware software. Although EL and impedance spectroscopy appeared to be effective, their use for the assessment of the cold hardiness is time consuming, also requiring expensive equipment and skilled staff. On the contrary, fractal analysis is a simple, objective and rapid method, also considering the cost of labor for collecting and scanning leaves plus processing the obtained information.

Measurements of Fv/Fm is a relatively sensitive parameter of the physiological status of chlorophyll and therefore often used to evaluate both cell damage and the response of the plant to cold stress [52]. Although artificial tests allow to evaluate many samples at the same time in a short period of time and sophisticated and precise freezing chamber systems are currently available, some uncertainties must be considered. In fact, in laboratory tests only the response of excised tissues is evaluated without considering the whole plant, it is not always possible to simulate the complex environmental conditions characterizing a natural frost episode. Furthermore, the duration and rate of artificial freezing can potentially affect the extent of the injury and the results obtained under artificial conditions do not always reflect the response or the re-growth of a species or cultivar under natural conditions. In addition, nucleation temperatures are lower in artificial freeze protocols than those observed under natural conditions, and therefore these conditions are not always reliable [63,120,158]. In addition, in vitro cultures techniques were used as laboratory freezing tests [114,163].

It is worth noting that many works were carried out in the 90–2000 s when genetic characterization in the olive tree still had little impact. Indeed, for example in Denney et al. [78] most of the varieties in the NCGR collection of Winters, CA were subsequently characterized and correctly identified by [76]. The lack of varietal authentication is one of the reasons for the contradictory results in the literature.

In recent years, and in particular under conditions of climate change, the duration of a freezing-event (“freezing-duration”) and the impact on plant survival is a debated issue [111]. A crucial aspect to consider is the experimental condition to which the plants are subjected. Several experimental assays were conducted under similar conditions where plant responses were assessed early, in the first 24–48 h, while transcriptomic work revealed a distinct early and long plant response. Leyva-Perez et al. [135] observed the gene expression response over 10 days and the trend of many genes were downregulated. This may indicate that further modifications and changes from a biochemical point of view have to be expected which probably have not yet been thoroughly investigated to date. However, there is a lack of consistent varietal screening with numerous tested varieties (in pots) associated with laboratory tests that certainly give a precise indication of the plant’s response from a physiological, biochemical and molecular point of view.

Notable advances have also been made with the use of high-resolution infrared thermography in our understanding of ice nucleation and propagation [64,164,165] and the properties of antifreeze proteins [166]. Despite these advances, significant improvements in plant cold hardiness have been elusive and problematic due to the complexity of this trait and its intimate connection to other plant developmental processes, especially growth and flowering. In addition, the relatively new field of epigenetics and epigenomics has demonstrated the key role that the environment can play on imprinting plant response to abiotic stress [166].

An integrated approach that takes into account the complexity of traits that contribute to plant cold hardiness will be needed to achieve advances that can be translated into practical solutions that address the challenges of a rapidly changing climate. In general, the correlation between a controlled freezing test and the results obtained in the field for several years, locations and for many parameters analyzed, could be an ideal test to effectively study cold tolerance and select resistant genotypes [44].

## 5. Agronomic Management for Frost Protection

The selection and use of tolerant cultivars and optimal area suitable for olive production are the first choices to limit freezing injuries. However, agronomic protection methods that have been developed can allow the recovery of a cold-damaged plant or reduce injury by making the plant colder tolerant.

In general, two main approaches are considered: (1) Recovery from freezing damage; (2) treatments to help the plant to cope with frost. The only treatment that allows the recovery of olive trees damaged by low temperatures is the cutting of the damaged parts (pruning) and to allow the development of new vegetation [13,42,73,85,167]. The authors suggested to intervene after having verified the extent of the damage (lightly damaged trees, moderately damaged trees, severely damaged trees) and preferably at the vegetative recovery (spring-summer). Plants with slightly chlorotic/brown leaves and reduced defoliation (23–25%) require no special pruning treatment. Recovery is usually rapid. Trees presenting moderate damage, (more marked defoliation, deep cracks on the 1- and 2-year-old branches) should undergo a regeneration pruning, allowing a rapid growth and a rapid reconstruction of the crown. Eventually, in trees in which the trunk and the aerial part are strongly compromised, the elimination of the entire crown or the uprooting of the tree are suggested [73,167]. To enhance and/or to acquire freezing tolerance in olive tree the exogenous application of physiologically active substances (i.e., growth regulators, nutrients) has been tested [19]. Fernández-Escobar et al. [168] evaluated nitrogen (N) influence on the frost tolerance of adult plants cv Picual. Leaves collected from plants with different nitrogen status (low, adequate, or high) were used in a controlled freezing test. Seasonal differences in response to low temperatures have been observed. In fact, an excess of N resulted in an increase in LT tolerance before dormancy (autumn), while in spring, an excess of the element made the plants more sensitive [168,169]. Treatments with copper (Cu) containing sprays, conducted during winter, made plants less sensitive to LT [19] and potassium (K) fertilization was recommended to reduce damage from low temperatures in the spring [169]. This could be related to the role of K in the regulation of osmotic and water potential [169]. Saadati and colleagues [170] revealed that foliar application of potassium solfate (K_2_SO_4_) at different concentrations, might play a key role in providing tolerance in olive plants. The obtained results in non-hardy Rashid cultivar highlighted an increase in protective compounds (carbohydrates, proline and total phenols) and in antioxidant enzyme activity, that may contrast the negative impact of cold stress. Foliar application of Mefluidine caused the increase of freezing tolerance in Frantoio frost-sensitive cultivar [43]. Authors postulated that synthetic growth regulator may activate a defensive response through the induction of water stress, the modification of cell solute content and the increase of biological membranes [43].

Many genes responsive to cold stress are also responsive to drought stress, suggesting the possible implications of water potential modifications for cold acclimation and frost-resistance in plants [171]. Water stress resulted in a significant increase in freezing tolerance determined, very probably, by decrease of tissue water content, osmotic adjustment and production of protective solutes [172]. Two studies [70,173] reported that water deficit significantly enhanced the olive freezing tolerance. Mild water stress and moderate water stress, before the onset of low temperatures (March- September) reduced the freezing injuries and increased freezing tolerance in Arbequina and in Barnea cvs [173], and in Arbequina, Changlot Real, Frantoio, Hojiblanca and Manzanilla cvs [70]. Water deficit induced the accumulation of osmotically active solutes and the decrease of RWC. These responses are involved in osmotic regulation, cryoprotection, and changes in the fluidity and stability of the cell membrane.

In addition, exogenous application of different phytohormones may reduce the harmful effects of low temperature. Exogenous application of salicylic acid (SA) [174] and jasmonates (JAs) and brassinosteroids (BRs) [175] have been used to improve plant tolerance and or resistance to low temperatures. SA is a growth regulator in plants and is an important signal molecule involved in plant responses to abiotic and biotic stresses. Three exogenous SA spray (0.5, 1, or 2 mM) and irrigation treatments (10 mL of SA solution) enhanced freezing tolerance in frost sensitive olive plant (cv Zard). The protective effect may be mediated via enzymatic activity (i.e., APX and SOD), and/or accumulation of compatible solute (i.e., proline) and phenolic compounds. Furthermore, the plants treated with SA (1.0 mM) showed a significantly lower ion leakage and MDA content [173]. Jasmonates (lipid-derived phytohormones,) and Brassinosteroids (steroid hormones) are novel phytohormones involved in plant developmental processes and plant adaptations to biotic and abiotic stresses [176,177]. Three different concentrations of methyl jasmonate (MeJA; 0.02, 0.1, 0.2 mM) and of 24–epibrassinolide (EBR; 0.2, 0.4, and 0.6 μM) respectively, were used to monitor the response to low temperatures in Koroneki cultivar [174]. Foliar application of MeJA and EBR improved FT of olive leaves by reduced ion-leakage and MDA content and increased proline, carbohydrates, total soluble protein and the total phenolic compounds. Eventually, phytohormone applications alleviated oxidative stress, increasing the levels of antioxidant enzymes [175]. In general, these studies, although preliminary, suggest that the treatments of olive trees with SA, JAs and BRs will maximize freezing tolerance of olive cultivars.

## 6. Conclusions and Future Perspectives

From the analysis of the many different research studies, considered in this review, it emerged that low temperatures, below the limits of adaptation, substantially influence the metabolism, viability, physiology, and productivity of olive tree. Furthermore, the severity of damage depends on the duration of event, the genotypes and plant stage of growth, and on the interactions with the environment, that can determine differences, between cultivars, in the response to the cold acclimation process, influencing their intrinsic tolerance to cold stress. One positive feature of the literature is that a wide range of cultivar has been studied, while a great lack is the understanding of the cold stress process in the open field, in fact most of the papers refer to observation in a protected environment. Although, over the years numerous researches have highlighted the morphological and physiological changes that occur following a freezing event, we are still far from knowing the different mechanisms responsible for tolerance to low temperatures in olive trees. The changes in the transcriptome of olive tree leaves exposed to CA suggest that olive tree activates known and conserved molecular responses to cold stress, such as induction of genes encoding enzymes associated to changes in membrane lipid composition, as well as abiotic stress related genes like dehydrins, the downregulation of photosynthesis-related genes, and the activation of the carbohydrate catabolism. However, specific peculiarities characterize the CA of olive tree, such as preferential osmolytes compounds, ROS scavenging systems and nuclear-organellar cross-talking.

A complete understanding of the physiological, biochemical and molecular mechanisms of tolerance in olive tree, with particular attention to the signaling pathways determining the response to low temperatures, will provide new breeding tools and help to select cultures with greater freezing tolerance.

In this review, some salient points on the current knowledge of damage, adaptation mechanisms and response to low temperatures in olive trees have been illustrated. The analysis of cold stress in olive trees is very complex and requires studies with multidisciplinary approaches that involve advanced researches on chromatin remodeling, epigenetic and epigenomic modifications, nucleus-organelle (chloroplasts and mitochondria) interactions and the subsequent use of synthetic biology for the control of gene expression in vivo. A great lack in the literature is the understanding of the cold stress process in the open field, in fact most of the papers refer to observation in a protected environment.

## Data Availability

Not applicable.

## Supplementary Materials

The following supporting information can be downloaded at: https://www.mdpi.com/article/10.3390/plants11101367/s1, Table S1: List of the most relevant frosts in some olive growing areas; Table S2: Evaluation of cold-hardiness and screening methods for freezing tolerance of olive cultivars. Refer to references [178,179,180,181,182,183,184,185,186,187,188,189,190,191,192,193,194,195,196,197,198,199,200,201] in supplementary materials.
