# Supplementary material for: Cold Stress, Freezing Adaptation, Varietal Susceptibility of Olea europaea L.: A Review
Source: Plants (Basel). 2022 May 20;11(10):1367. doi: 10.3390/plants11101367 (PMC9144808; doi:10.3390/plants11101367)
Supplement: Supplementary file 1 [file plants-11-01367-s001.zip › Supplementary 1.pdf]

Table S1. List of the most relevant frosts in some olive growing areas.

Year	Temp. °C	Cultivation area	Source
**1216**		Lombardia, Tuscany, Italy:	Trinci 1763
**1234**		Parma, Italy	Fabbri 2017
**1424-1431**		Veneto, Italy:	Zorzi 2001
**1432**		Pordenone, Italy:	Zamparutti, 2004
**1450**		Tuscany, Italy:	Tavanti 1819
**1455**		Tuscany, Italy:	Baldini, 2003
**1476-1480**		Tuscany, Italy:	Tavanti 1819
**1490**		Veneto Cividale-UD), Italy	Battigelli, 2002, Zorzi 2001
**1510**		Tuscany (FI), Italy	Trinci 1763
**1514**		Veneto Italy	Zorzi 2001
**1564**		Italy	Tavanti 1819
**1568**		Umbria (Montefalco-PG), Italy	Panbuffetti 1956
**1587**		Tuscany, Italy:	Baldini, 2003
**1600**		Tuscany, Italy:	Presta, 1794; Trinci 1763
**1608**		Tuscany, Italy:	Baldini, 2003, Tavanti 1819
**1610**		Tuscany, Italy:	Tavanti 1819
**1664**		Tuscany, Italy:	Tavanti 1819
**1709-**	-13°C	Tuscany (PI, LI, GR); Lombardia (MN); Emilia (PR), Veneto, Italy; Perpignan-Nizza, France	Pellini, 1996, Morettini 1961, Mariti 1797, Caruso 1882, Pecori 1889, Presta 1794,
**1747-1770**		Tuscany, Italy:	Morettini 1961, Tavanti 1819
**1749-55-60-66-67-68**		Tuscany, Veneto, Italy	Tavanti 1819, Zorzi 2001
**1782**		Greece	Xoplaki et al., 2001
**1788-89**		Tuscany, Veneto, Italy	Presta 1794, Giovine 1788, Morettini 1961, Zorzi 2001
**1807/08**		Greece	Xoplaki et al., 2001
**1794-1812-1816**		Veneto, Italy	Zorzi 2001
**1846-47**	-13°C	Tuscany,Italy	Ridolfi, 1847; Cuppari 1848, Pecori 1889
**1849**	-20°C	Tuscany, Italy	Caruso 1882, Pecori 1889
**1855**	-18°C	Montpellier, France	Martins in Pecori 1889
**1870**	-15/16°C	Montpellier, Nemes, France	Martins in Pecori 1889
**1871-72**	-11°C	Tuscany, Italy	Presta 1794, Caruso 1882, Pecori 1889
**1893**	-11°C	Tuscany, Italy	Morettini 1961
**1895**	-10.6°C	Tuscany, Italy	Morettini 1961
**1907**	-11.7°C	Tuscany, Italy	Morettini 1961
**1929-'30**	-22°C	Tuscany, Umbria Italy	Brunetti, 2001,Morettini1961
**1939-40**	-15°C	Tuscany, Italy	Breviglieri 1940
**1956**	-20.1°C	Tuscany, Umbria, Italy:	Scaramuzzi Andreucci 1957, Morettini 1961, Morettini Marinucci Jacoponi 1956
**1967/8**	-10.4°C	Umbria, Italy	Fontanazza, Preziosi, 1967
**1983-1985-1987**		Turkey	Ozturk et al., 2021
**1985-**	-23°C	Tuscany, Italy	Scaramuzzi et al 1989
**1990**	-9.4/-22.8 °C	California, USA	Denney et al., 1993
**1991/1992**		Crete, Greece	Xoplaki et al., 2001
**2000/2005/2007/2008**		Saudi Arabia	Naser et al., 2018

**2010**	-1.7/-5°C	New South Wales, Victoria, Australia	Sergeeva, 2010
**2012**	-4.7/6.4°C	Marche, Italy	Lodolini et al, 2016
**2016**	-15°C	Golestan Iran	Karamatlou et al., 2019
**2018**	4/-6°C	Apulia, Italy	Paradiso, 2018

Supplementary 1 References

Baldini, E.. *Notizie sull'olivicoltura Bolognese*. Ed. Accademia Nazionale di Ggricoltura. Bologna, Italy, 2003.

Breviglieri, N. Osservazioni sui danni causati all'Olivo dalle basse temperature dell'inverno 1939-40 nel Mugello. *L'Olivicoltore* **1940**, Roma, Italy.

Caruso, G. Monografia dell'olivo. In *Enciclopedia Agraria Italiana*,. UTET Ed., Torino, Italy, 1882; Volume 3 (5), pp. 501-533

Cuppari, P. Alcune osservazioni intorno agli effetti del gelo sugli olivi. *Bullettino agrario*, , **1848.** 5,XXII.

Denney, J.O.; McEachern, G.R. 1983. An analysis of several climatic temperature variables dealing with olive reproduction. *J. Amer. Soc. Hort. Sci*. **1983,** 108, 578-581

Fabbri, A. The olive in Northern Italy. A Mediterranean tale. Rivista di Storia dell'Agricoltura 2017, LVII (1) 1-32.

Francolini, F. *Olivicoltura*. Un. Tip. Ed. Torinese, Torino, Italy, 1923.

Fontanazza G.; Preziosi P. L'olivo e le basse temperature. Osservazioni su 37 cultivar da olio e 20 cultivar da mensa. *L'Italia Agricola* **1969**, 7-8, 737-745.

Gaetani; L. I danni del gelo degli olivi nel 1929 in Umbria. *La Metereologia pratica* **1938**

Giovine G.M., 1788. Relazione del danno cagionato agli olivi della campagna di Molfetta dalla gelata del 30-31 dicembre 1788. Ed. *Opere*, vol II.

Denney, J.0. AccoFding to NCDC records, serious freezes in which temperatures dipped below 20°F (-6.7°C) occurred at several sites in 1913,1919, 1930,1932,1937,1948,1949,1950,1972, and 1978. Of the freezes before 1990, the 1913 and 1932 freezes were the most severe and extensive

Lodolini, E.M.; Alfei, B.; Santinelli, A.; Cioccolanti, T.; Polverigiani, S; Neri, D. Frost tolerance of 24 olive cultivars and subsequent vegetativere-sprouting as indication of recovery ability. Sci Hort 2016, 211 (1), 152–157. https://doi.org/10.1016/j.scienta.2016.08.025

Karamatlou, I.; Navabpour, S.; Zainilnejad, Kh.; Tavakol, E.; Hosseini Mazinani, M. Morphological Evaluation and Selection of Tolerant Trees to Freezing Stress at the Olive Orchards in Golestan Province. *Journal of Horticultural Science* **2019** 33, 287-299. ISSN: 2008 - 4730

Mariti, G. *L'Odeporico, ossia Itinerario per le colline pisane*, Firenze 1797, pp.. 54.

Morettini, A. Sulla ricostruzione degli olivi daneggiati dalle basse temperature del 1956. In Ricostruzione degli olivi danneggiati dal freddo. Accademia dei Georgofili Ed.; Firenze, Italy, 1989; Volume 2, pp. 1-42.

Morettini, A; Marinucci, M.; Jacoboni, N. Olivi colpiti dal gelo. REDA Ed., Roms, Italy, 1965; pp. 69.

Naser, I.; Hermogino, R.; Angeles, C:; Abu Kashem A. Effect of frost and salts dissolved after heavy rain on the productivity of olive trees under desert growing conditions. *Journal of Agriculture and Allied Sciences* **2018**, RRJAAS| 7 (1), 85-103.

Panbuffetti; P. 1956. Ieri come oggi. La galaverna del. *Agricoltore* **1568**, n. 15.

Pellini, U. *Alberi nella storia di Reggio.* AGE Ed.Reggio Emilia, Italy, 1996.

Ozturk, M.; Altay, V.; Gönenç, T.M.; Unal, B.T.; Efe, R.; Akçiçek, E.; Bukhari, A. An Overview of Olive Cultivation in Turkey: Botanical Features, Eco-Physiology and Phytochemical Aspects. *Agronomy* **2021**, 11, 295. https://doi.org/10.3390/agronomy11020295

Paradiso, N. Attenzione ai danni da gelo su olivo in Puglia. *L'Informatore Agrario* **2018**, 16, 74-75.

Pecori, R.. *La coltura dell'olivo in Italia* Ricci M Ed.; Florence, Italy, 1889; pp. 428. .

Presta, G. 1786. Memoria su i saggi diversi di olio e su della ragia di ulivo della penisola salentina. In *Memoria intorno a sessantadue saggi diversi di olio,* 1nd ed.; GS Romano Ed. Lecce Italy, 1786; Volume I, pp. 1-42.

Presta, G. *Degli ulivi, delle ulive e della maniera di cavar l'olio*. Stamperia Reale, Ed.a Italy, 1794; Volume II pp. 619

Ridolfi, C. Del danno provato dagli olivi pel gelo del dicembre 1846. *Giornale Agrario Toscano* **1847**, XXI, 71-731847

Scaramuzzi, F.; Andreucci. E. Indagini e osservazioni sui danni provocati dalle nime termiche del febbraio 1956 agli olivi nei vivai di Pescia. Nuovo Giornale Botanico Italiano 1957, 64, 19-104.
Scaramuzzi, F.; Andreucci, E. Indagini e osservazioni sui danni provocati dalle minime termiche del febbraio 1956 agli olivi nei vivai di Pescia. *Nuovo Giorn. Bot. It.*, **1957,** 66(1-2): 19-124.
Sergeeva, V. Frost and chilling injuries in olive. Australian and New Zealand. *Olive grower and Processor* **2010**, 74, 23-34.
Tavanti, G. . *Trattato Teorico-Pratico Completo Sull'Ulivo*. Piatti Ed. Firenze, italy; 1819; pp. 259, Italy.
Trinci, C. 1763. Trattato degli Ulivi. In *L'Agricoltore sperimentato, ovvero Regole Generali sopra l'agricoltura, 7 nd* G. Dorigoni Ed.; Venezia, Italy, 1763, Volume 1, pp. 526
Xoplaki, E.; Panagiotis Maheras, P; Juerg Luterbacher, J. Variability of climate in meridional balkans During the periods 1675–1715 and 1780–1830 And its impact on human life. *Climatic Change* **2001,** 48, 581–615
Zamparotti, P. 2004 L'inverno più freddo degli ultimi 500 anni. *Meteo Clima e Storia* **2004.**
Zorzi A. 2001. La Repubblica del Leone. Bompiani Ed. pp. 766. Italy
